# CRISPR/Cas9‐mediated Genome‐editing Reveals 10 Testis‐enriched Genes and One Non‐testis‐enriched Gene are Dispensable for Male Fecundity in Mice

**DOI:** 10.1111/andr.70144

**Published:** 2025-11-10

**Authors:** Yumiao Qiu, Keisuke Shimada, Masahito Ikawa

**Affiliations:** ^1^ Research Institute for Microbial Diseases The University of Osaka Osaka Japan; ^2^ Graduate School of Medicine The University of Osaka Osaka Japan; ^3^ The Institute of Medical Science The University of Tokyo Tokyo Japan; ^4^ Center For Infectious Disease Education and Research The University of Osaka Osaka Japan

**Keywords:** CRISPR/Cas9, knockout mice, male infertility, spermatozoa, testis

## Abstract

**Background:**

More than 1000 genes have been identified as predominantly expressed in the human testis. Advances in gene editing technologies have enabled the rapid and efficient generation of genetically engineered mice. This approach facilitates the screening of genes essential for spermatogenesis by analyzing knockout mouse models.

**Objectives:**

This study aimed to elucidate the essential genes in male reproductive function by generating knockout mouse models.

**Materials and Methods:**

We selected 11 target genes that may have potential roles in the male reproductive system based on a public database. Knockout mouse lines of these target genes were generated using the CRISPR/Cas9 system to elucidate their functions in male reproduction. Also, we conducted natural mating tests to elucidate fecundity and analyzed the phenotype of the knockout males.

**Results:**

Natural mating tests revealed that all 11 gene‐deficient mouse lines maintained normal male fertility. The phenotypic analysis, including testis appearance and weight, histology of testis and epididymis, and sperm motility and morphology, showed no apparent abnormalities.

**Discussion and Conclusion:**

These results suggest that each gene is not essential for male reproductive function.

## 1 Introduction

Spermatogenesis is an extremely complex physiological process involving the sequential differentiation of various spermatogenic cells within the seminiferous tubules. This process includes three successive stages: spermatogonial stem cells proliferate through mitosis, spermatocytes undergo meiosis, and spermatids undergo morphological differentiation and elongation to form mature spermatozoa.^1^, ^2^, ^3^ Disruption at any stage of the spermatogenesis process can result in male infertility. To ensure continuous sperm production, precise coordination and regulation of the sequential stages of spermatogenesis are essential.^4^

Spatial human proteome analysis has identified over 1000 genes predominantly expressed in the testis.^5^ These testis‐enriched genes may play significant roles in spermatogenesis and sperm function in humans. Since genes are highly conserved between humans and mice,^6^ generating knockout (KO) mouse models for these genes and analyzing their phenotype is suitable for offering clear evidence whether or not a gene of interest is crucial for male fertility. These results provide valuable insight into the potential physiological roles and functions of orthologous genes in humans. Our laboratory employed the CRISPR/Cas9 system to generate KO mouse models lacking testis‐enriched genes and revealed that many of these genes are indispensable for male infertility. For instance, TSKS,^7^ SPATA33,^8^ and CCDC188^9^ have been identified as critical proteins for spermiogenesis, while NICOL^10^ and NELL2^11^ play essential roles in epididymal sperm maturation. Additionally, IZUMO1^12^ and TMEM81^13^ have been demonstrated to be required for spermatozoa‐egg interaction. On the other hand, our previous studies also identified that many testis‐enriched genes were individually dispensable for male fertility.^14^, ^15^, ^16^, ^17^, ^18^, ^19^ These results suggest that not all of these testis‐enriched genes are essential for male fertility, and other testis‐enriched genes may compensate for their functions in male reproduction. Due to the absence of an in vitro system capable of producing fully functional spermatozoa so far,^20^ generating KO mouse models using the CRISPR/Cas9 system is the gold standard for discovering essential genes for male fertility, and it provides valuable insights into their physiological functions.

In this study, we selected ten testis‐enriched genes (*1700016H13Rik*, *1700031M16Rik*, *Atp6v1e2*, *Ccdc185*, *Ccdc81*, *Or2ag2b*, *P3r3urf*, *Pbp2*, *Prdx6b*, and *Zfp474*) and one non‐testis‐enriched gene (*Adamts15*). We then generated these gene KO mouse models one by one using the CRISPR/Cas9 genome editing system to investigate their functions in the male reproductive system. Phenotypic analyses of these KO mouse models demonstrated that these 11 genes are individually dispensable for male fertility in mice.

## 2 Materials and Methods

### 2.1 Animals

All animal experiments were approved by the Animal Care and Use Committee of the Research Institute for Microbial Diseases, the University of Osaka. Animals were housed in a temperature‐controlled environment with 12 h light cycles and free access to food and water. B6D2F1 (C57BL/6 × DBA2) and ICR mice were used as embryo donors and foster mothers, respectively. These animals were purchased from CLEA Japan, Inc. (Tokyo, Japan) or Japan SLC (Shizuoka, Japan).

### 2.2 Digital Polymerase Chain Reaction

The mRNA expression patterns of target mouse genes and their orthologous human genes by tissue or by cell type were determined using MRGDv2 (https://orit.research.bcm.edu/MRGDv2).^21^

### 2.3 Generation of KO Mice

All KO mouse lines in this study were generated with the CRISPR/Cas9 system as previously performed.^22^ We designed guide RNAs (gRNAs) to remove all or most of the coding sequence. CRISPRdirect software^23^ was used to avoid off‐target possibilities. Synthesized crRNAs (Merck, Darmstadt, Germany), tracrRNA (Merck), and CAS9 protein (Thermo Fisher Scientific, Waltham, MA, USA) were incubated to make the CAS9 ribonucleoprotein (RNP) complex. The obtained RNP complex was electroporated into fertilized eggs using a NEPA21 electroporator (NEPA GENE, Chiba, Japan). Fertilized eggs that had been electroporated were transplanted into the oviducts of pseudopregnant females. The obtained pups were genotyped by polymerase chain reaction (PCR) and then subjected to Sanger sequencing to verify the deleted sequence.

### 2.4 Genotyping Analysis

Genotyping PCR was performed using KOD FX Neo (Toyobo, Osaka, Japan). The primers used in this study are listed in Table S1.

### 2.5 In Vivo Male Fertility Test

To confirm the fertility of KO male mice, natural mating tests were conducted. Three male mice were individually caged with three B6D2F1 females for two months. Male mice were removed after the mating period, and females were kept for another three weeks to count the final litters. Both plug and pup numbers were checked at approximately 10 AM every weekday to determine the number of copulations and litter size.

### 2.6 Morphological and Histological Analysis

Male mice were euthanized, and the cauda epididymides were dissected. Spermatozoa were collected from the cauda epididymis and suspended in Toyoda, Yokoyama, Hoshi (TYH) medium.^24^ A sperm suspension was mounted on MAS‐coated glass slides (Matsunami Glass, Osaka, Japan), and a cover slip (Matsunami) was added. Sperm morphology was observed using a BX53 microscope (Olympus, Tokyo, Japan).

Morphological and histological analyses of the testis and epididymis were conducted as previously described.^25^ Male mice were euthanized, and the testes and epididymides were dissected. After measuring the testicular weight, the testes and epididymides were fixed with Bouin's fixative (Polysciences, Warrington, PA, USA). Fixed testes and epididymides were embedded in paraffin, sectioned, rehydrated, and treated with 1% periodic acid for 10 min, followed by treatment with Schiff's reagent (Merck) for 20 min. The sections were stained with Mayer's hematoxylin solution (Fujifilm Wako, Osaka, Japan) prior to imaging and observed using a BX53 microscope. The number of germ cells (spermatogonia, spermatocytes, round spermatids, and elongated spermatids) was counted at stage VII seminiferous tubule.

### 2.7 Sperm Motility Analysis

Sperm motility analysis was conducted as described previously.^8^ Cauda epididymal spermatozoa were suspended and incubated in TYH medium, which can induce sperm capacitation.^24^ Sperm motility was then measured using the CEROS II sperm analysis system (software version 1.5; Hamilton Thorne Biosciences, Beverly, MA, USA). The motility of epididymal spermatozoa was recorded after 10 min and 2 h of incubation in TYH medium.

### 2.8 Statistical Analyses

Statistical analyses were performed using a two‐tailed Student's t‐test or One‐way ANOVA (* *p* < 0.05, ** *p* < 0.01) by GraphPad Prism 9.5.1 (GraphPad, San Diego, CA, USA). Data represent the means ± standard deviation (SD).

## 3 Results

### 3.1 Expression Patterns of 11 Genes in Mice and Humans

**FIGURE 1:**
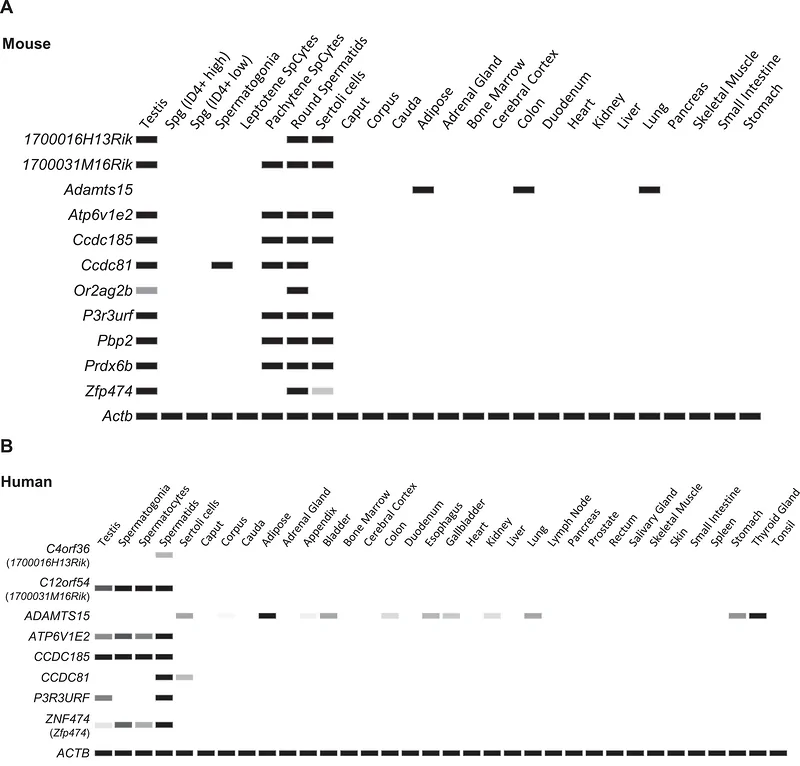
Expression patterns of target genes in various tissues. (A, B) Digital PCR depicting the transcripts per million (TPM) value per tissue per gene from published mouse and human RNA‐seq datasets.^21^, ^44^, ^45^, ^46^ Indifferentiation 4 (ID4) plays an important role in the regulation of self‐renewal by spermatogonial stem cells.^47^ Spg (ID4+ high) indicates spermatogonia with a high expression level of ID4, and Spg (ID4+ low) indicates spermatogonia with a low expression level of ID4. SpCytes indicate spermatocytes. (A) Gene expression patterns in mouse tissues. *Actb* was used as a loading control. (B) Gene expression patterns in human tissues. *ACTB* was used as a loading control.

To investigate the tissue expression profiles of the 11 genes in this study, we conducted digital PCR using the database.^21^ *1700016H13Rik*, *1700031M16Rik*, *Atp6v1e2*, *Ccdc185*, *Ccdc81*, *Or2ag2b*, *P3r3urf*, *Pbp2*, *Prdx6b*, and *Zfp474* are testis‐enriched genes; however, *Adamts15* is expressed in adipose, colon, and lung (Figure 1A). Then, we checked the expression patterns of their human orthologs. Since *Or2ag2b* (paralog of *Or2ag2*), *Prdx6b* (paralog of *Prdx6*), and *Pbp2* are not conserved in humans, the expression patterns of eight human ortholog genes were confirmed with digital PCR. Our results indicate that *C4orf36* (ortholog of *1700016H13Rik*), *C12orf54* (ortholog of *1700031M16Rik*), *ATP6V1E2*, *CCDC185*, *CCDC81*, *P3R3URF*, and *ZNF474* (ortholog of *Zfp474*) are predominantly expressed in the testis and/or germ cells, while *ADAMTS15* is expressed in various tissues and cell types (Figure 1B). Based on these results, we selected these 10 testis‐enriched genes (*1700016H13Rik*, *1700031M16Rik*, *Atp6v1e2*, *Ccdc185*, *Ccdc81*, *Or2ag2b*, *P3r3urf*, *Pbp2*, *Prdx6b*, and *Zfp474*) and one non‐testis‐enriched gene (*Adamts15*) as target genes to generate KO mouse lines.

### 3.2 Generation of KO Mice

To explore the in vivo function of these target genes in male reproduction, 11 KO mouse lines were generated individually using the CRISPR/Cas9 system. The guide RNA/Cas9 RNP complex was electroporated into two‐pronuclear stage embryos. Each gene‐KO mouse line was successfully established by deleting most or all of the protein‐coding region. The efficiency of embryo transfer ranged from 3.6% to 36.3%, and CRISPR/Cas9 efficiency varied from 10.0% to 69.2%. Details of gene editing efficiency using the zygote electroporation method and mutation patterns of each gene‐KO mouse line are summarized in Table S2.

### 3.3 Fertility Tests for KO Male Mice

To evaluate fecundity, each KO male mouse was housed with three wild‐type (WT) female mice for eight weeks. Male mice were removed from females and kept for another three weeks. For comparison, three WT males were tested in parallel as positive controls. The average litter size of each KO male was approximately 7.6–9.8 pups per litter, which was comparable to the WT controls (9.1 ± 2.8 pups per litter) (Table 1). These results indicate that all KO mouse lines examined in this study maintained normal fertility.

**TABLE 1:** Male fertility of 11 mutant mouse lines.

Gene symbol	Official full name	Genotype	Average litter size	Number of males	Number of deliveries	Number of pups	Number of plugs	Mating period (week)
Wild type	–	–	9.1 ± 2.8	3	26	237	27	8+3
*1700016H13Rik*	RIKEN cDNA 1700016H13 gene	−6243/‐6243	8.8 ± 2.0	5	32	283	41	8+3
*1700031M16Rik*	RIKEN cDNA 1700031M16 gene	−18,283/‐18,283	8.9 ± 2.9	3	19	170	25	8+3
*Adamts15*	ADAM metallopeptidase with thrombospondin type 1 motif 15	−20,198/‐20,198	9.2 ± 3.1	3	18	166	23	8+3
*Atp6v1e2*	ATPase, H+ transporting, lysosomal V1 subunit E2	−787/‐787	9.1 ± 3.3	3	21	191	26	8+3
*Ccdc185*	coiled‐coil domain containing 185	−1708/‐1708	8.2 ± 2.8	3	20	164	22	8+3
*Ccdc81*	coiled‐coil domain containing 81	−37,018/‐37,018	9.0 ± 2.7	3	20	179	28	8+3
*Or2ag2b*	olfactory receptor family 2 subfamily AG member 2B	−1095/‐1095	9.4 ± 3.0	3	23	217	23	8+3
*P3r3urf*	Pik3r3 upstream reading frame	−91/‐91	7.6 ± 2.4	3	22	168	26	8+3
*Pbp2*	phosphatidylethanolamine binding protein 2	−455/‐455	9.3 ± 2.4	3	22	205	28	8+3
*Prdx6b*	peroxiredoxin 6B	−846/‐846	9.6 ± 3.0	3	22	211	27	8+3
*Zfp474*	zinc finger protein 474	−1004/‐1004	9.8 ± 2.2	3	23	226	26	8+3

### 3.4 Phenotypic Analysis of *Atp6v1e2*, *Ccdc81*, and *Prdx6b* KO Mouse Lines

We performed phenotypic analyses to investigate whether these male mice have abnormalities in spermatogenesis and sperm functions. The analyses of *Atp6v1e2*, *Ccdc81*, and *Prdx6b* KO males will be described herein. In contrast, those of *1700016H13Rik*, *1700031M16Rik*, *Adamts15*, *Ccdc185*, *Or2ag2b*, *P3r3urf*, *Pbp2*, and *Zfp474* KO males are presented in Figures S1–S8.

**FIGURE 2:**
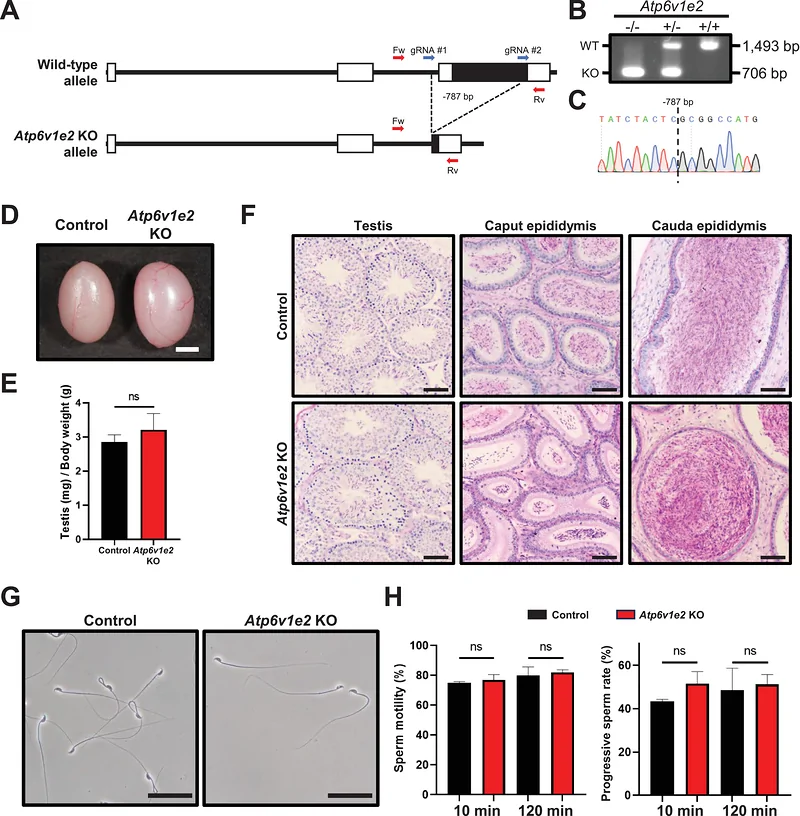
Phenotypic analysis of *Atp6v1e2* knockout (KO) male mice. (A) KO strategy for generating *Atp6v1e2* KO mice. The upper and bottom panels show diagrams for WT and KO alleles, respectively. Two gRNAs (blue arrows) were designed to remove almost the whole open reading frame. Fw and Rv are primers for genotyping. Our study generated an *Atp6v1e2* KO mouse line with a 787‐bp deletion. (B) Genotyping of *Atp6v1e2* KO mutant mice. Fw/Rv primers shown in Figure 2A were used. (C) Sanger sequence of *Atp6v1e2* KO mutant mice. The dashed line marks the deletion breakpoint. (D) Gross morphology of control and *Atp6v1e2* KO testes. Scale bar: 2.0 mm. (E) The average testis weight per body weight of control and *Atp6v1e2* KO mice (ns indicates not significant, Student's t‐test, *N* = 3). (F) Hematoxylin and PAS‐stained sections of the testes, caput, and cauda epididymides. Scale bars: 50 µm. (G) Morphology of spermatozoa from control and *Atp6v1e2* KO mice obtained from the cauda epididymis. Scale bars: 50 µm. (H) Motile sperm rate and progressive sperm rate from control and *Atp6v1e2* KO mice. Motility was checked after 10 min and 2 h of incubation in Toyoda, Yokoyama, Hoshi (TYH) media (ns indicates not significant, Student's t‐test, *N* = 3).

To generate the *Atp6v1e2* KO mouse line, two gRNAs were designed to remove most of the protein‐coding region (Figure 2A). Using the primers presented in Figure 2A, genomic PCR was performed (Figure 2B). Sanger sequence confirmed the KO mouse line has a 787‐bp deletion (Figure 2C). *Atp6v1e2* KO mice are viable and do not show any overt abnormalities. Morphological and histological analysis of the testes of *Atp6v1e2* KO mice revealed that there were no significant differences in gross appearance (Figure 2D), testicular weights (Figure 2E), testicular histology (Figure 2F), or germ cell number (Figure S9A–D) between control and *Atp6v1e2* KO mice. Similarly, histological analyses of the caput and cauda epididymides showed no significant differences between control and KO (Figure 2F). To examine the effects of ATP6V1E2 absence on sperm development, we observed sperm morphology and found no overt differences (Figure 2G). Furthermore, sperm motility was evaluated using computer‐assisted sperm analysis (CASA), which indicated that sperm motility, progressive sperm rate, and the other motility parameters in *Atp6v1e2* KO mice were comparable to those of control mice (Figure 2H and Table S4).

**FIGURE 3:**
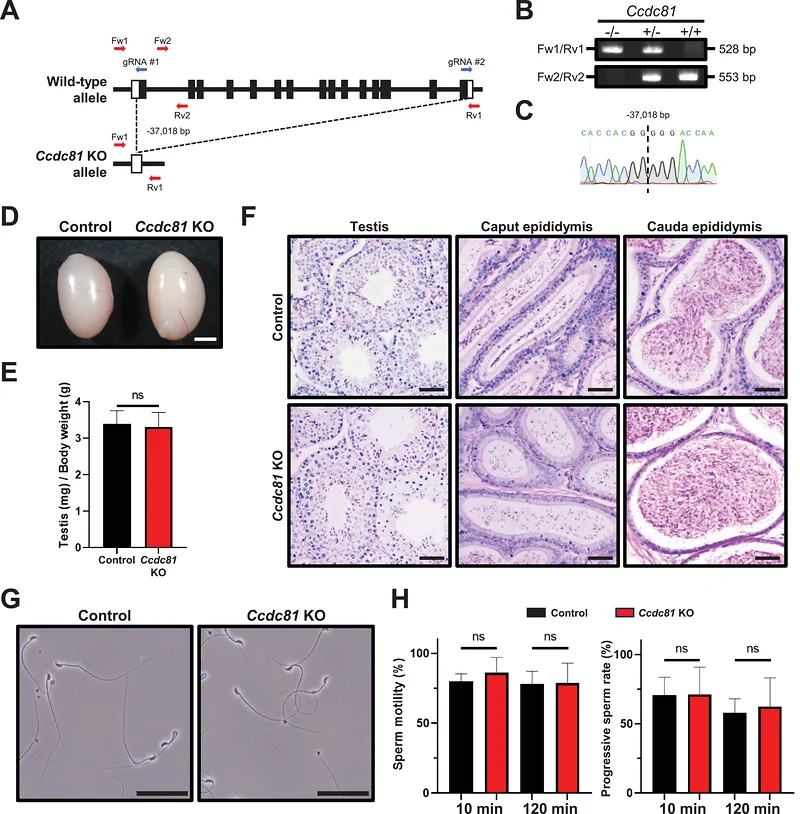
Phenotypic analysis of *Ccdc81* knockout (KO) male mice. (A) KO strategy for generating *Ccdc81* KO mice. The upper and bottom panels show diagrams for WT and KO alleles, respectively. Two gRNAs (blue arrows) were designed to target exons 1 and 15. Fw1 and Fw2 are forward primers for genotyping. Rv1 and Rv2 are reverse primers for genotyping. Our study generated a *Ccdc81* KO mouse line with a 37,018‐bp deletion. (B) Genotyping of *Ccdc81* KO mutant mice. Fw1/Rv1 and Fw2/Rv2 primers shown in Figure 3A were used. (C) Sanger sequence of *Ccdc81* KO mutant mice. The dashed line marks the deletion breakpoint. (D) Gross morphology of control and *Ccdc81* KO testes. Scale bar: 2.0 mm. (E) The average testis weight per body weight of control and *Ccdc81* KO mice (ns indicates not significant, Student's t‐test, *N* = 3). (F) Hematoxylin and PAS‐stained sections of the testes, caput, and cauda epididymides. Scale bars: 50 µm. (G) Morphology of spermatozoa from control and *Ccdc81* KO mice obtained from the cauda epididymis. Scale bars: 50 µm. (H) Motile sperm rate and progressive sperm rate from control and *Ccdc81* KO mice. Motility was checked after 10 min and 2 h of incubation in Toyoda, Yokoyama, Hoshi (TYH) media (ns indicates not significant, Student's t‐test, *N* = 3).

*Ccdc81* KO mice were generated by designing two gRNAs to remove the whole open reading frame of *Ccdc81*. After electroporation and subsequent mating, we obtained a mutant mouse line harboring a 37,018‐bp deletion (Figure 3A–C). *Ccdc81* KO mice were viable, and no overt abnormalities were found. *Ccdc81* KO testis showed a normal gross appearance, and testis weights relative to body weights were comparable to those of control mice (Figure 3D,E). Histological analyses showed no abnormality in the histology of testes and epididymides, and the number of germ cells in *Ccdc81* KO male mice (Figure 3F and Figure S9A–D). Moreover, normal sperm morphology could be observed in *Ccdc81* KO male mice (Figure 3G). Analysis of sperm motility using CASA demonstrated no significant differences between control and KO male mice (Figure 3H and Table S4).

**FIGURE 4:**
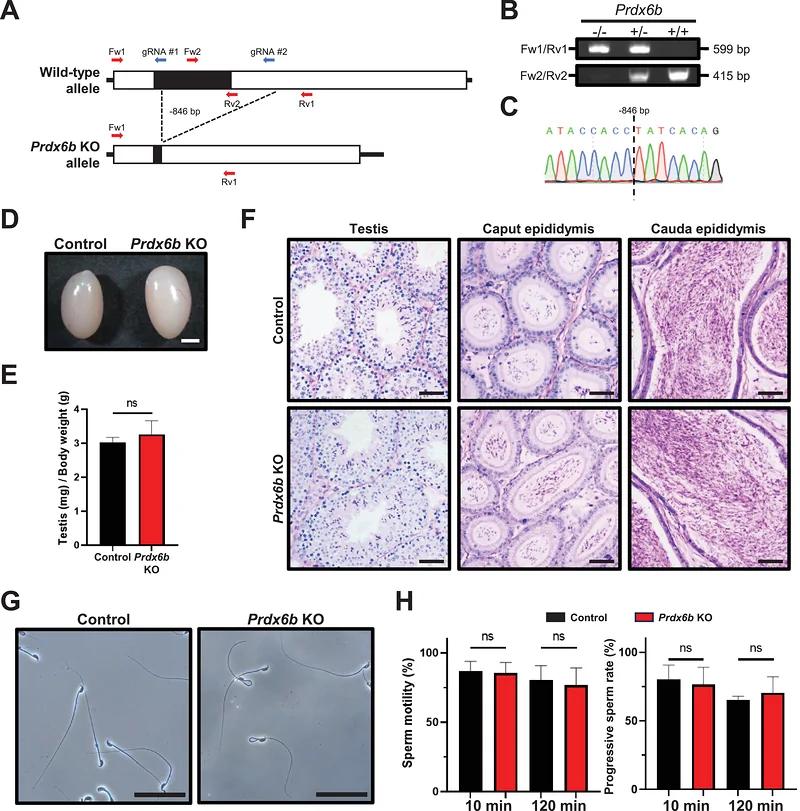
Phenotypic analysis of *Prdx6b* knockout (KO) male mice. (A) KO strategy for generating *Prdx6b* KO mice. The upper and bottom panels show diagrams for WT and KO alleles, respectively. Two gRNAs (blue arrows) were designed to remove almost the whole open reading frame. Fw1 and Fw2 are forward primers for genotyping. Rv1 and Rv2 are reverse primers for genotyping. Our study generated a *Prdx6b* KO mouse line with an 846‐bp deletion. (B) Genotyping of *Prdx6b* KO mutant mice. Fw1/Rv1 and Fw2/Rv2 primers shown in Figure 4A were used. (C) Sanger sequence of *Prdx6b* KO mutant mice. The dashed line marks the deletion breakpoint. (D) Gross morphology of control and *Prdx6b* KO testes. Scale bar: 2.0 mm. (E) The average testis weight per body weight of control and *Prdx6b* KO mice (ns indicates not significant, Student's t‐test, *N* = 3). (F) Hematoxylin and PAS‐stained sections of the testes, caput, and cauda epididymides. Scale bars: 50 µm. (G) Morphology of spermatozoa from control and *Prdx6b* KO mice obtained from the cauda epididymis. Scale bars: 50 µm. (H) Motile sperm rate and progressive sperm rate from control and *Prdx6b* KO mice. Motility was checked after 10 min and 2 h of incubation in Toyoda, Yokoyama, Hoshi (TYH) media (ns indicates not significant, Student's t‐test, *N* = 3).

Similarly, *Prdx6b* was knocked out by targeting its coding region with two gRNAs. Mutant offspring bearing an 846‐bp deletion were obtained, and their genotyping was identified by genomic PCR (Figure 4A–C). Phenotypic analyses demonstrated that *Prdx6b* KO males exhibited normal testicular appearance and weight (Figure 4D,E), normal testicular and epididymal histology (Figure 4F), comparable germ cell number (Figure S9A–D), and normal sperm morphology (Figure 4G) and motility (Figure 4H and Table S4).

## 4 Discussion

Within the seminiferous tubules of the testes, spermatozoa develop through a highly organized process known as spermatogenesis. Due to the challenges in replicating spermiogenesis in vitro so far,^26^ the functional analysis of genes involved in mammalian spermiogenesis has primarily relied on KO mouse models.^27^ In this study, we examined the physiological functions of potential target proteins involved in male fertility using KO mouse models.

Through in silico analysis, *1700016H13Rik*, *1700031M16Rik*, *Atp6v1e2*, *Ccdc185*, *Ccdc81*, *Or2ag2b*, *P3r3urf*, *Pbp2*, *Prdx6b* and *Zfp474* were identified as testis‐enriched genes in mice (Figure 1A). Even though *Adamts15* exhibited high expression levels in lung, colon, and adipose tissue (Figure 1A), a previous study has reported that the protein levels of ADAMTS1 and ADAMTS5 were lower in the semen of infertile patients.^28^ Given that ADAMTS15 belongs to the same protein family and shares a similar domain structure with ADAMTS1 and ADAMTS5,^29^, ^30^ we hypothesized that it may play a role in the male reproductive system. In this paper, we selected these 11 genes to generate KO mouse models to assess their potential involvement in male fertility. However, all KO male mice showed normal fecundity, indicating that these 11 genes are individually dispensable for male fertility (Table 1).

Based on previous studies, no information is available regarding the roles of *1700016H13Rik*, *1700031M16Rik*, *Adamts15, Ccdc185*, *Or2ag2b*, *P3r3urf*, *Pbp2*, and *Zfp474* in the male reproductive system. This study revealed that these genes were not essential for male fecundity in mice. Besides, the functions of the remaining genes will be discussed below.

ATPase, H+ transporting, lysosomal V1 subunit E2 (ATP6V1E2) is a component of a spermatozoa‐specific V‐ATPase protein, which is expressed in secretory acrosomes.^31^, ^32^ In rats, ATP6V1E2 plays a critical role in acrosome acidification, which is essential for fertilization.^33^ Similarly, sperm localization analysis of human samples indicated that ATP6V1E2 localized to the acrosome vesicle and was strongly correlated with fertilization rates.^34^ However, our findings reveal that *Atp6v1e2* KO male mice are fertile (Table 1). These results may indicate that ATP6V1E2 might function differently among species and/or that other proteins may compensate for the functions of ATP6V1E2 in mice.

Coiled‐coil domain containing 81 (CCDC81) is a member of the CCDC family, characterized by proteins with coiled‐coil domains that contain two to six helices.^35^ Based on the mass spectrometry of sperm centrioles, CCDC81 has been identified as a centrosome‐associated protein.^36^ Additionally, CCDC81 protein has been found to associate with centrosome organization in protein‐protein interaction and may function as a potential cargo‐binding protein in coordination with Dynein‐VII.^37^ Therefore, it was hypothesized that *Ccdc81* KO spermatozoa may show low sperm motility with defects in the sperm neck region, but there were no significant differences in fertility and sperm motility between *Ccdc81* KO mice and control mice (Table 1, Figure 3H, and Table S4).

Peroxiredoxin 6B (PRDX6B) is a member of the peroxiredoxins (PRDXs) family. These family proteins were localized to various spermatozoa compartments, including the head, mitochondrial sheath, and flagellum.^38^, ^39^ Gong et al. reported that spermatozoa from infertile patients exhibited reduced levels of PRDX6, which was associated with low motility, higher levels of lipid peroxidation, and DNA damage.^40^ Additionally, other studies have demonstrated that *Prdx6* KO mice produce fewer offspring than controls.^41^ In this paper, we investigated *Prdx6b*, a paralog gene of *Prdx6*, which shows testis‐enriched expression (Figure 1A). Different from PRDX6, fertility was not affected by the loss of PRDX6B (Table 1), despite its mRNA expression in the testis (Figure 1A). Because these paralogous proteins may have similar functions, they may compensate for each other. Therefore, *Prdx6* and *Prdx6b* double KO mice may show severe abnormalities compared to *Prdx6* single KO mice in the male reproductive system.

While KO mouse lines in this study displayed no obvious defects in fertility, we cannot exclude the possibility that these genes contribute to male reproductive function in humans. For instance, we revealed that loss of ATP6V1E2 did not affect male fertility in mice (Figure 2). However, Cozzubbo et al. demonstrated a strong association between ATP6V1E2 expression in spermatozoa and fertilization outcomes in patients undergoing ICSI.^34^ Therefore, ATP6V1E2 should have some important functions in the male reproductive system in humans. One reason that ATP6V1E2 works differently in humans and mice may be due to functional redundancy. The V‐ATPase V1 complex, also referred to as ATP6V1,^42^ is composed of multiple subunits (A–H),^43^ and these paralogs may work to complement the function of ATP6V1E2. Another example involves members of the PRDX6 family, PRDX6 and PRDX6B. Disruption of each gene individually does not significantly affect male fertility^41^ since they may compensate for each other. To overcome this problem, it may be necessary to generate multiple gene KO animals, thereby minimizing compensatory effects from paralogs and more accurately revealing their contributions to reproductive function.

In summary, we applied the CRISPR/Cas9 system to disrupt 10 testis‐enriched genes and one non‐testis‐enriched gene individually in mice. Although our mating results reveal that not all of these genes are essential for male fecundity in mice, these findings contribute to the efficient allocation of research resources by preventing redundant efforts on non‐essential genes. Furthermore, this study facilitates the prioritization of future research toward identifying genes that play critical roles in male reproduction.

## Author Contributions

**Y.Q**., **K.S**., and **M.I**. designed the research; **Y.Q**. and **K.S**. performed the research and analyzed the data; **Y.Q**., **K.S**., and **M.I**. wrote the manuscript. All authors read, corrected, and approved the manuscript.

## Funding

This work was supported by the Ministry of Education, Culture, Sports, Science and Technology/Japan Society for the Promotion of Science KAKENHI grants (JP23K05831 to K.S., and JP21H05033 to M.I.); Takeda Science Foundation grants to K.S.; the Senri Life Science Foundation grant to K.S.; the Eunice Kennedy Shriver National Institute of Child Health and Human Development (R01HD088412 to M.I.); Japan Science and Technology Agency, Support for Pioneering Research Initiated by the Next Generation (JST SPRING) Grant Number JPMJSP2138 to Y.Q.

## Conflicts of Interest

The authors declare no conflicts of interest.

## Supporting information




**Supporting Information File 1**: andr70144‐sup‐0001‐SuppMat.pdf.

## Data Availability

Frozen spermatozoa from all KO males were deposited at both the Riken BioResource Center (RBRC), Ibaraki, Japan, and the Center for Animal Resources and Development (CARD), Kumamoto University, Kumamoto, Japan. The detailed information is listed in Table S3. KO mice are available through these centers.
