# Supplementary material for: CRISPR/Cas9‐mediated Genome‐editing Reveals 10 Testis‐enriched Genes and One Non‐testis‐enriched Gene are Dispensable for Male Fecundity in Mice
Source: Andrology. 2025 Nov 10;14(7):2150–9. doi: 10.1111/andr.70144 (PMC12961275; doi:10.1111/andr.70144)
Supplement: Supplementary file 1 [file ANDR-14-2150-s001.pdf]

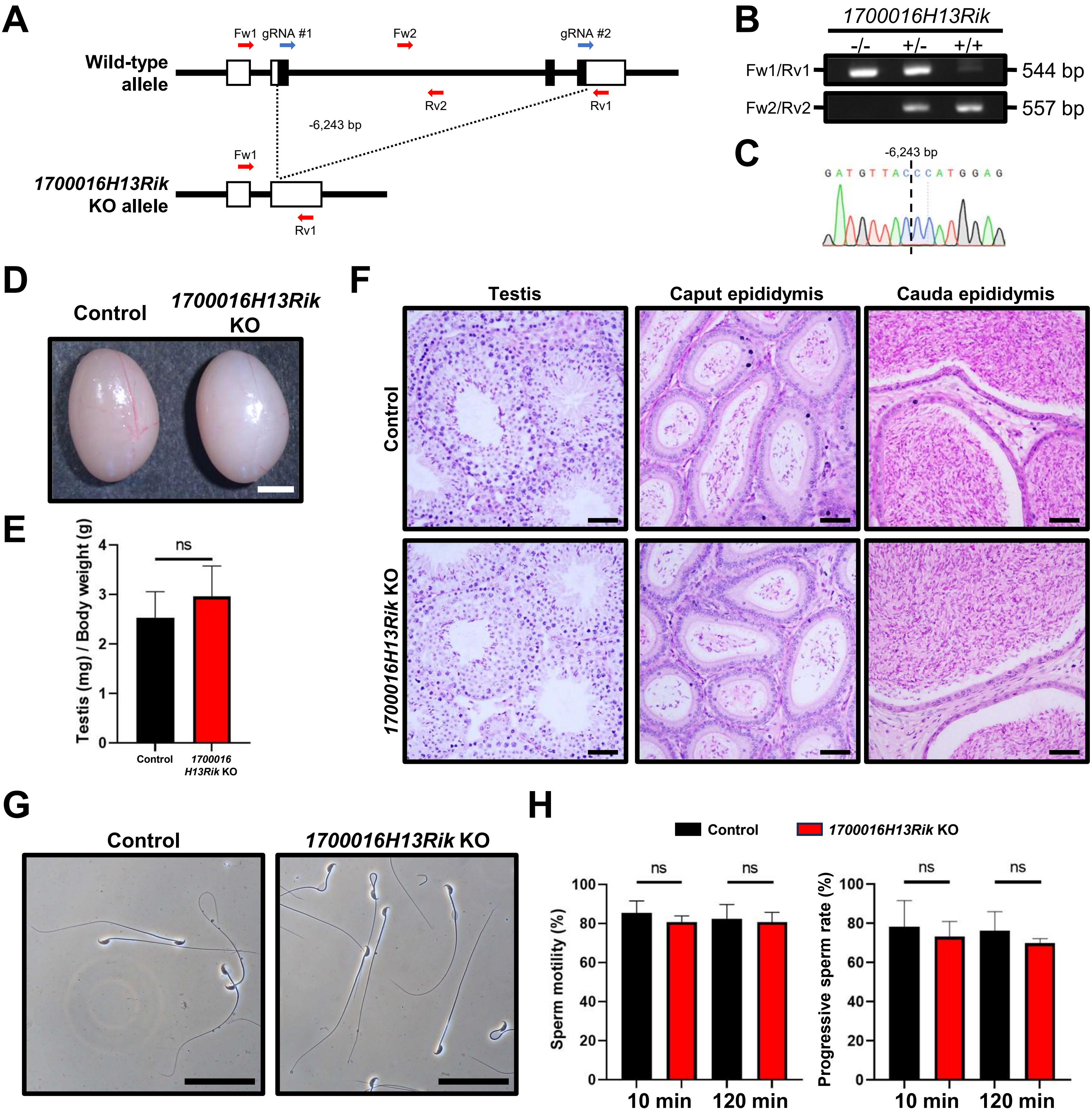

Figure S1. Phenotypic analysis of *1700016H13Rik* KO male mice.
(A) KO strategy for generating *1700016H13Rik* KO mice. The upper and bottom panels show diagrams for WT and KO alleles, respectively. Two gRNAs (blue arrows) were designed to target exons 2 and 4. Fw1 and Fw2 are forward primers for genotyping. Rv1 and Rv2 are reverse primers for genotyping. Our study generated a *1700016H13Rik* KO mouse line with a 6,243-bp deletion. (B) Genotyping of *1700016H13Rik* KO mutant mice. Fw1/Rv1 and Fw2/Rv2 primers shown in Fig. S1A were used. (C) Sanger sequence of *1700016H13Rik* KO mutant mice. The dashed line marks the deletion breakpoint. (D) Gross morphology of control and *1700016H13Rik* KO testes. Scale bar: 2.0 mm. (E) The average testis weight per body weight of control and *1700016H13Rik* KO mice (ns indicates not significant, Student's t-test, $N = 3$). (F) Hematoxylin and PAS-stained sections of the testes, caput, and cauda epididymides. Scale bars: 50 µm. (G) Morphology of spermatozoa from control and *1700016H13Rik* KO mice obtained from the cauda epididymis. Scale bars: 50 µm. (H) Motile sperm rate and progressive sperm rate from control and *1700016H13Rik* KO mice. Motility was checked after 10 min and 2 h of incubation in TYH media (ns indicates not significant, Student's t-test, $N = 3$).

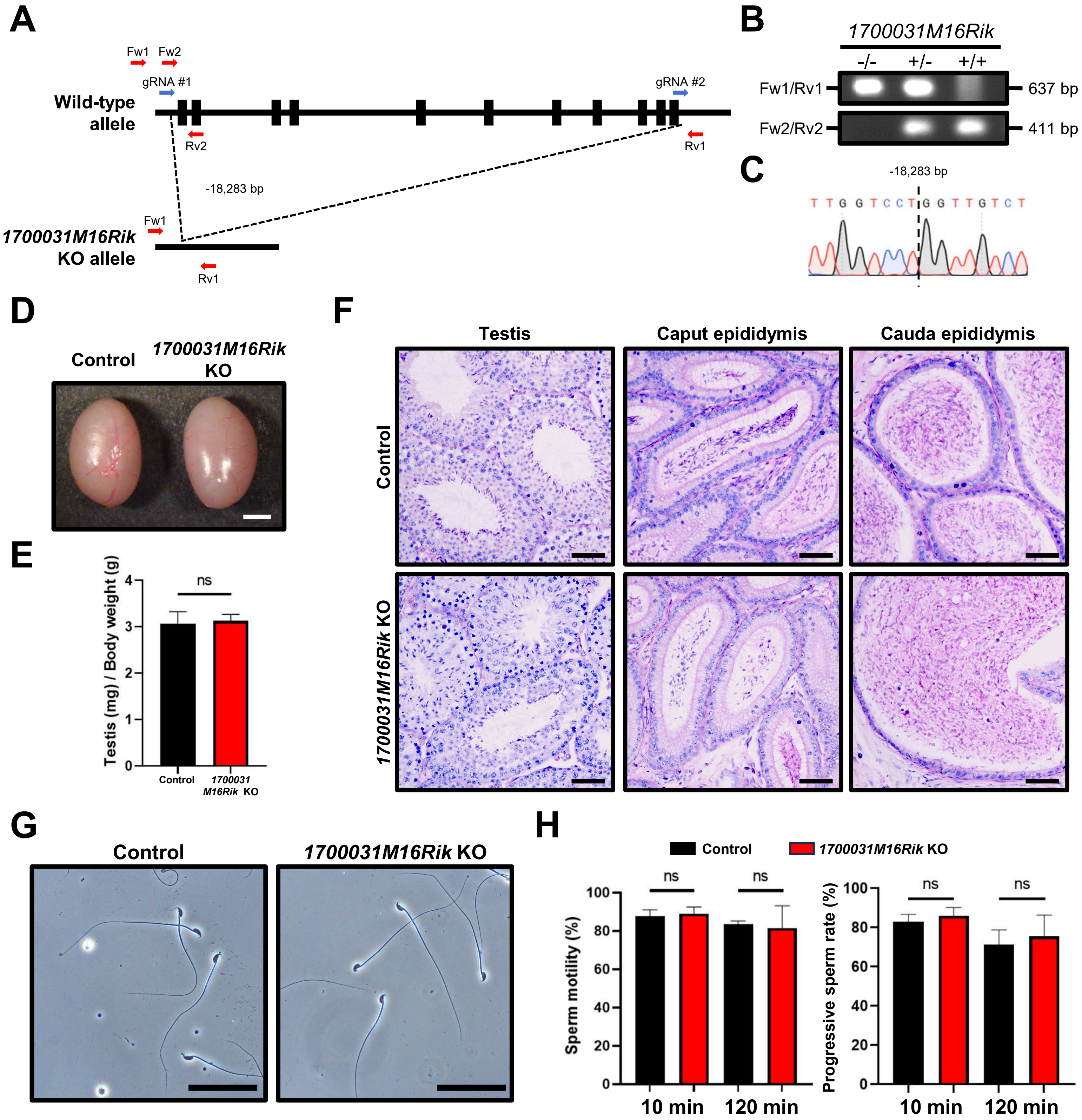

Figure S2. Phenotypic analysis of *1700031M16Rik* KO male mice.
(A) KO strategy for generating *1700031M16Rik* KO mice. The upper and bottom panels show diagrams for WT and KO alleles, respectively. Two gRNAs (blue arrows) were designed to target to remove the whole open reading frame. Fw1 and Fw2 are forward primers for genotyping. Rv1 and Rv2 are reverse primers for genotyping. Our study generated a *1700031M16Rik* KO mouse line with an 18,283-bp deletion. (B) Genotyping of *1700031M16Rik* KO mutant mice. Fw1/Rv1 and Fw2/Rv2 primers shown in Fig. S2A were used. (C) Sanger sequence of *1700031M16Rik* KO mutant mice. The dashed line marks the deletion breakpoint. (D) Gross morphology of control and *1700031M16Rik* KO testes. Scale bar: 2.0 mm. (E) The average testis weight per body weight of control and *1700031M16Rik* KO mice (ns indicates not significant, Student's t-test, *N* = 3). (F) Hematoxylin and PAS-stained sections of the testes, caput, and cauda epididymides. Scale bars: 50 µm. (G) Morphology of spermatozoa from control and *1700031M16Rik* KO mice obtained from the cauda epididymis. Scale bars: 50 µm. (H) Motile sperm rate and progressive sperm rate from control and *1700031M16Rik* KO mice. Motility was checked after 10 min and 2 h of incubation in TYH media (ns indicates not significant, Student's t-test, *N* = 3).

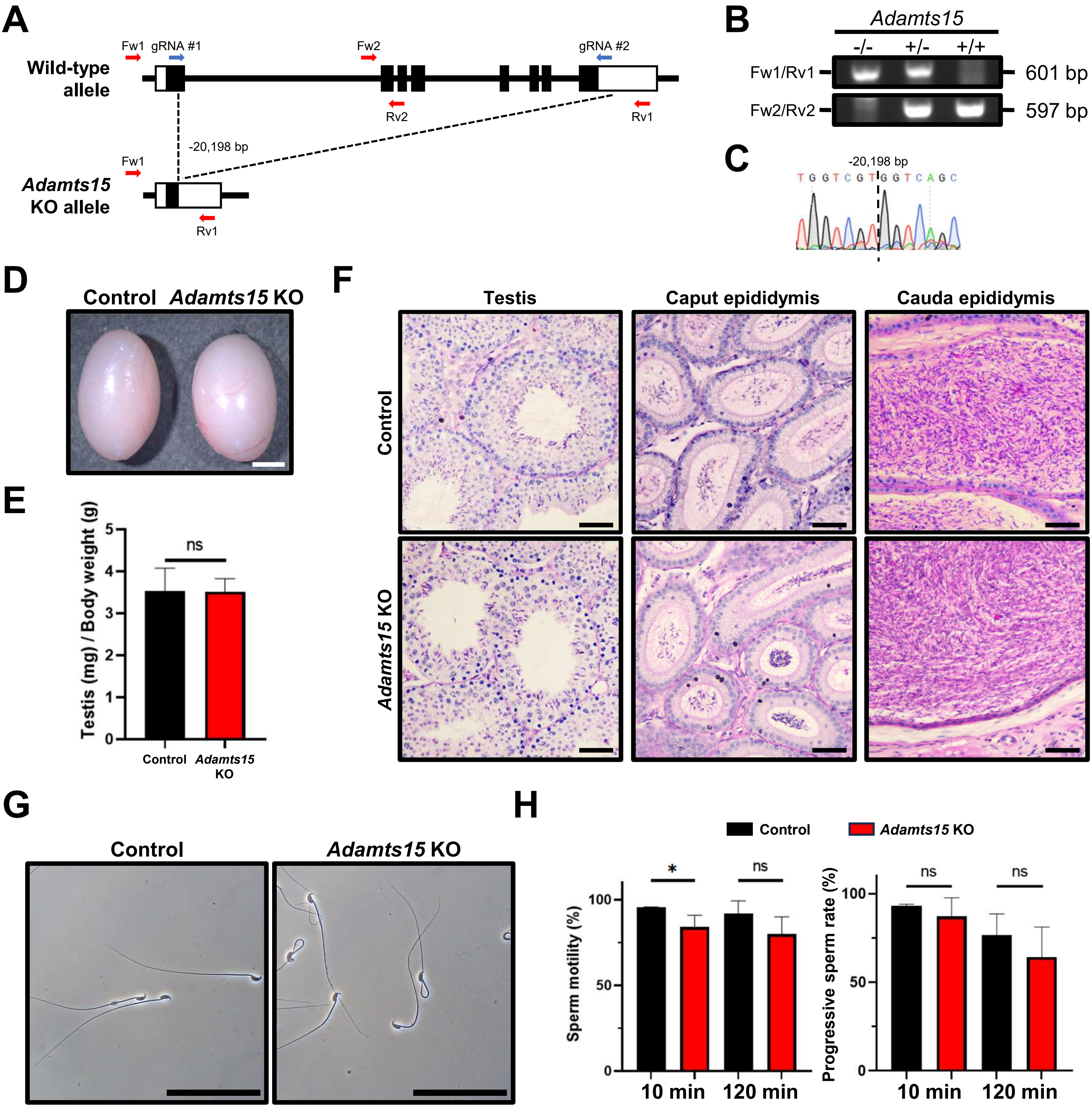

Figure S3. Phenotypic analysis of *Adamts15* KO male mice.
(A) KO strategy for generating *Adamts15* KO mice. The upper and bottom panels show diagrams for WT and KO alleles, respectively. Two gRNAs (blue arrows) were designed to target exons 1 and 8. Fw1 and Fw2 are forward primers for genotyping. Rv1 and Rv2 are reverse primers for genotyping. Our study generated an *Adamts15* KO mouse line with a 20,198-bp deletion. (B) Genotyping of *Adamts15* KO mutant mice. Fw1/Rv1 and Fw2/Rv2 primers shown in Fig. S3A were used. (C) Sanger sequence of *Adamts15* KO mutant mice. The dashed line marks the deletion breakpoint. (D) Gross morphology of control and *Adamts15* KO testes. Scale bar: 2.0 mm. (E) The average testis weight per body weight of control and *Adamts15* KO mice (ns indicates not significant, Student's t-test, $N = 3$). (F) Hematoxylin and PAS-stained sections of the testes, caput, and cauda epididymides. Scale bars: 50 µm. (G) Morphology of spermatozoa from control and *Adamts15* KO mice obtained from the cauda epididymis. Scale bars: 50 µm. (H) Motile sperm rate and progressive sperm rate from control and *Adamts15* KO mice. Motility was checked after 10 min and 2 h of incubation in TYH media (* $P < 0.05$, ns indicates not significant, Student's t-test, $N = 3$).

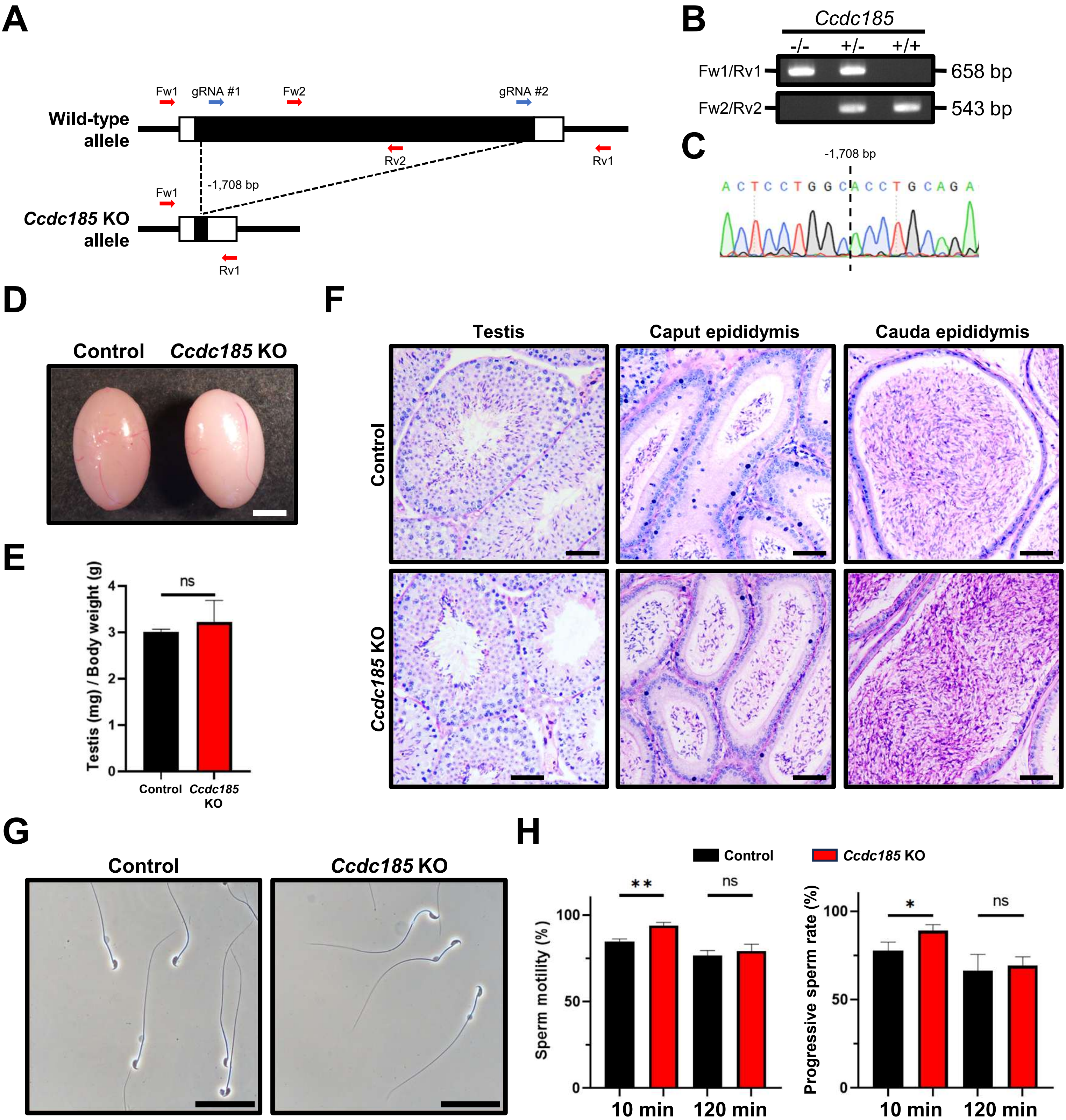

Figure S4. Phenotypic analysis of *Ccdc185* KO male mice.
(A) KO strategy for generating *Ccdc185* KO mice. The upper and bottom panels show diagrams for WT and KO alleles, respectively. Two gRNAs (blue arrows) were designed to remove almost the whole open reading frame. Fw1 and Fw2 are forward primers for genotyping. Rv1 and Rv2 are reverse primers for genotyping. Our study generated a *Ccdc185* KO mouse line with a 1,708-bp deletion. (B) Genotyping of *Ccdc185* KO mutant mice. Fw1/Rv1 and Fw2/Rv2 primers shown in Fig. S4A were used. (C) Sanger sequence of *Ccdc185* KO mutant mice. The dashed line marks the deletion breakpoint. (D) Gross morphology of control and *Ccdc185* KO testes. Scale bar: 2.0 mm. (E) The average testis weight per body weight of control and *Ccdc185* KO mice (ns indicates not significant, Student's t-test, $N = 3$). (F) Hematoxylin and PAS-stained sections of the testes, caput, and cauda epididymides. Scale bars: 50 µm. (G) Morphology of spermatozoa from control and *Ccdc185* KO mice obtained from the cauda epididymis. Scale bars: 50 µm. (H) Motile sperm rate and progressive sperm rate from control and *Ccdc185* KO mice. Motility was checked after 10 min and 2 h of incubation in TYH media (* $P < 0.05$, ** $P < 0.01$, ns indicates not significant, Student's t-test, $N = 3$).

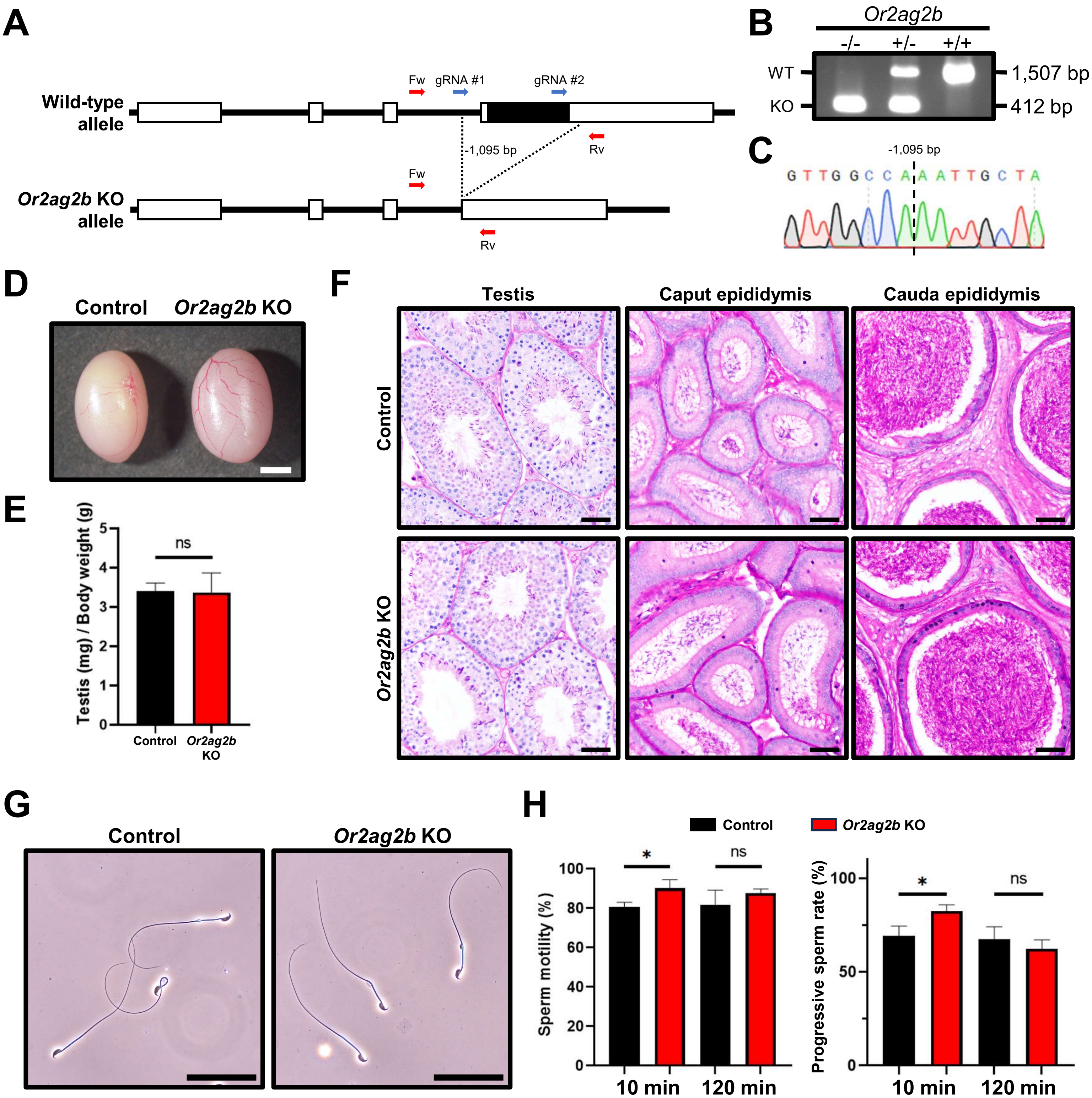

Figure S5. Phenotypic analysis of *Or2ag2b* KO male mice.
(A) KO strategy for generating *Or2ag2b* KO mice. The upper and bottom panels show diagrams for WT and KO alleles, respectively. Two gRNAs (blue arrows) were designed to remove the whole open reading frame. Fw and Rv are primers for genotyping. Our study generated an *Or2ag2b* KO mouse line with a 1,095-bp deletion. (B) Genotyping of *Or2ag2b* KO mutant mice. Fw/Rv primers shown in Fig. S5A were used. (C) Sanger sequence of *Or2ag2b* KO mutant mice. The dashed line marks the deletion breakpoint. (D) Gross morphology of control and *Or2ag2b* KO testes. Scale bar: 2.0 mm. (E) The average testis weight per body weight of control and *Or2ag2b* KO mice (ns indicates not significant, Student's t-test, $N = 3$). (F) Hematoxylin and PAS-stained sections of the testes, caput, and cauda epididymides. Scale bars: 50 µm. (G) Morphology of spermatozoa from control and *Or2ag2b* KO mice obtained from the cauda epididymis. Scale bars: 50 µm. (H) Motile sperm rate and progressive sperm rate from control and *Or2ag2b* KO mice. Motility was checked after 10 min and 2 h of incubation in TYH media (* $P < 0.05$, ns indicates not significant, Student's t-test, $N = 3$).

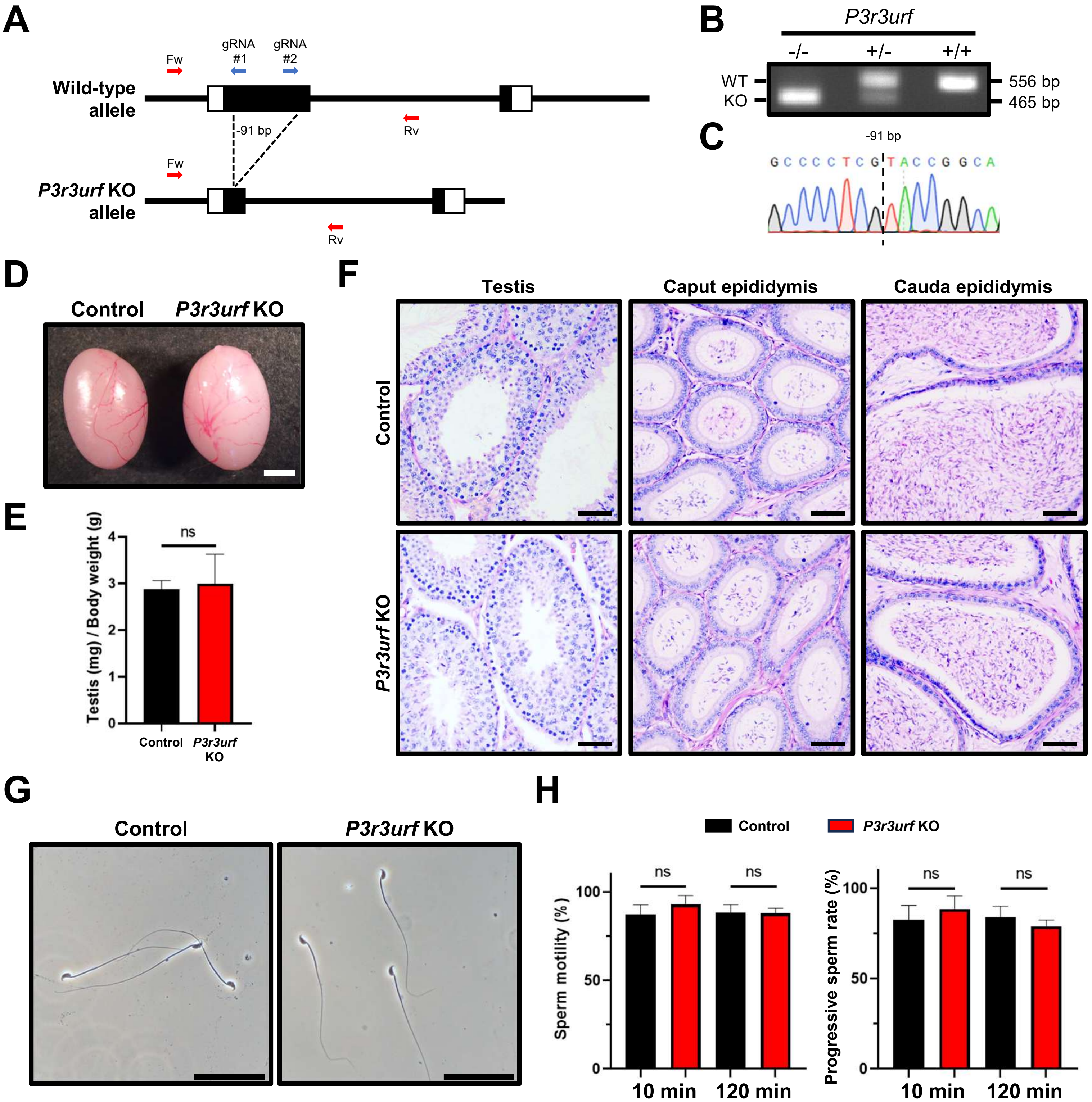

Figure S6. Phenotypic analysis of *P3r3urf* KO male mice.
(A) KO strategy for generating *P3r3urf* KO mice. The upper and bottom panels show diagrams for WT and KO alleles, respectively. Two gRNAs (blue arrows) were designed to target exon 1. Fw and Rv are primers for genotyping. Our study generated a *P3r3urf* KO mouse line with a 91-bp deletion. (B) Genotyping of *P3r3urf* KO mutant mice. Fw/Rv primers shown in Fig. S6A were used. (C) Sanger sequence of *P3r3urf* KO mutant mice. The dashed line marks the deletion breakpoint. (D) Gross morphology of control and *P3r3urf* KO testes. Scale bar: 2.0 mm. (E) The average testis weight per body weight of control and *P3r3urf* KO mice (ns indicates not significant, Student's t-test, *N* = 3). (F) Hematoxylin and PAS-stained sections of the testes, caput, and cauda epididymides. Scale bars: 50 µm. (G) Morphology of spermatozoa from control and *P3r3urf* KO mice obtained from the cauda epididymis. Scale bars: 50 µm. (H) Motile sperm rate and progressive sperm rate from control and *P3r3urf* KO mice. Motility was checked after 10 min and 2 h of incubation in TYH media (ns indicates not significant, Student's t-test, *N* = 3).

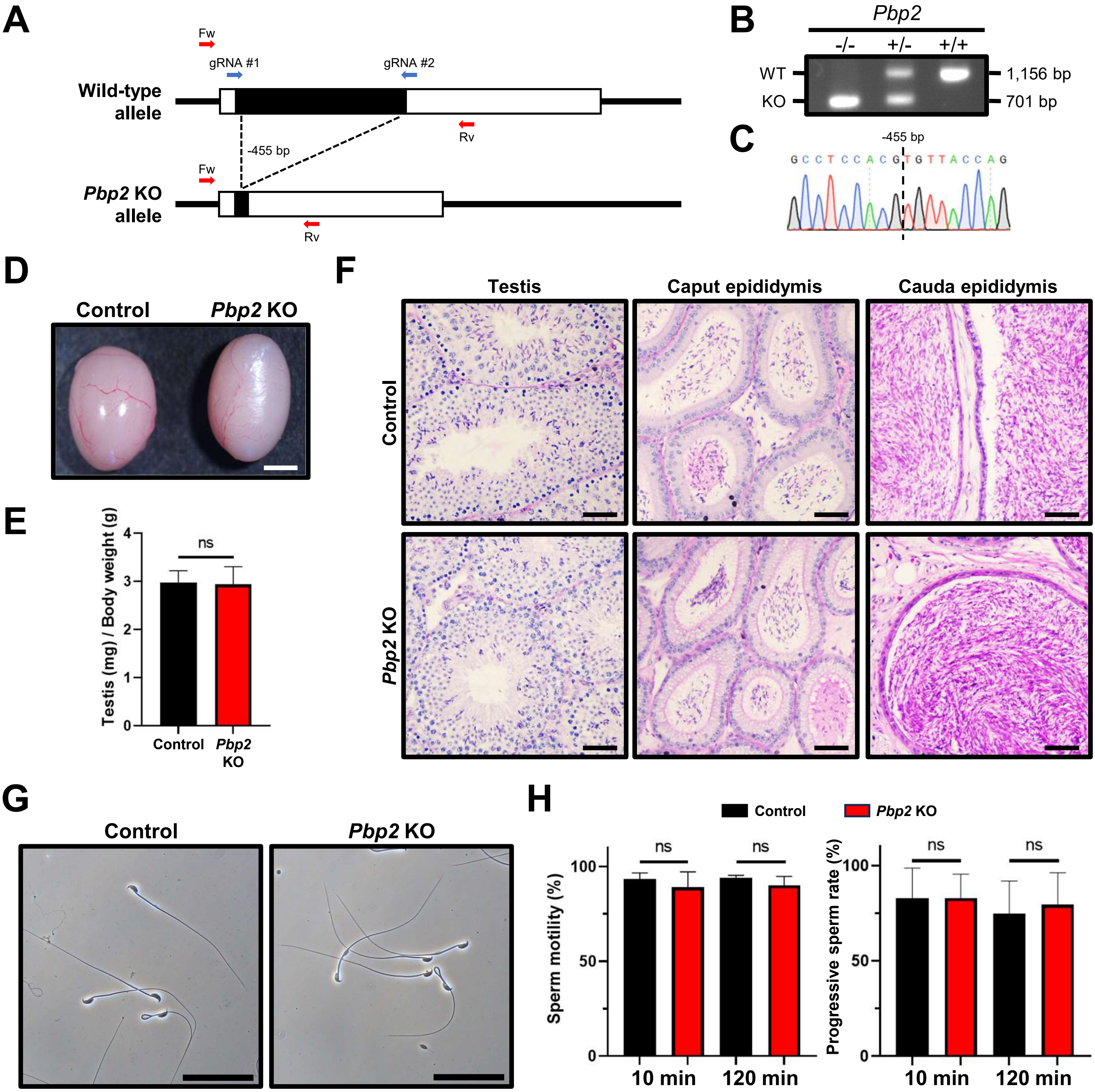

Figure S7. Phenotypic analysis of *Pbp2* KO male mice.
(A) KO strategy for generating *Pbp2* KO mice. The upper and bottom panels show diagrams for WT and KO alleles, respectively. Two gRNAs (blue arrows) were designed to remove almost the whole open reading frame. Fw and Rv are primers for genotyping. Our study generated a *Pbp2* KO mouse line with a 455-bp deletion. (B) Genotyping of *Pbp2* KO mutant mice. Fw/Rv primers shown in Fig. S7A were used. (C) Sanger sequence of *Pbp2* KO mutant mice. The dashed line marks the deletion breakpoint. (D) Gross morphology of control and *Pbp2* KO testes. Scale bar: 2.0 mm. (E) The average testis weight per body weight of control and *Pbp2* KO mice (ns indicates not significant, Student's t-test, *N* = 3). (F) Hematoxylin and PAS-stained sections of the testes, caput, and cauda epididymides. Scale bars: 50 µm. (G) Morphology of spermatozoa from control and *Pbp2* KO mice obtained from the cauda epididymis. Scale bars: 50 µm. (H) Motile sperm rate and progressive sperm rate from control and *Pbp2* KO mice. Motility was checked after 10 min and 2 h of incubation in TYH media (ns indicates not significant, Student's t-test, *N* = 3).

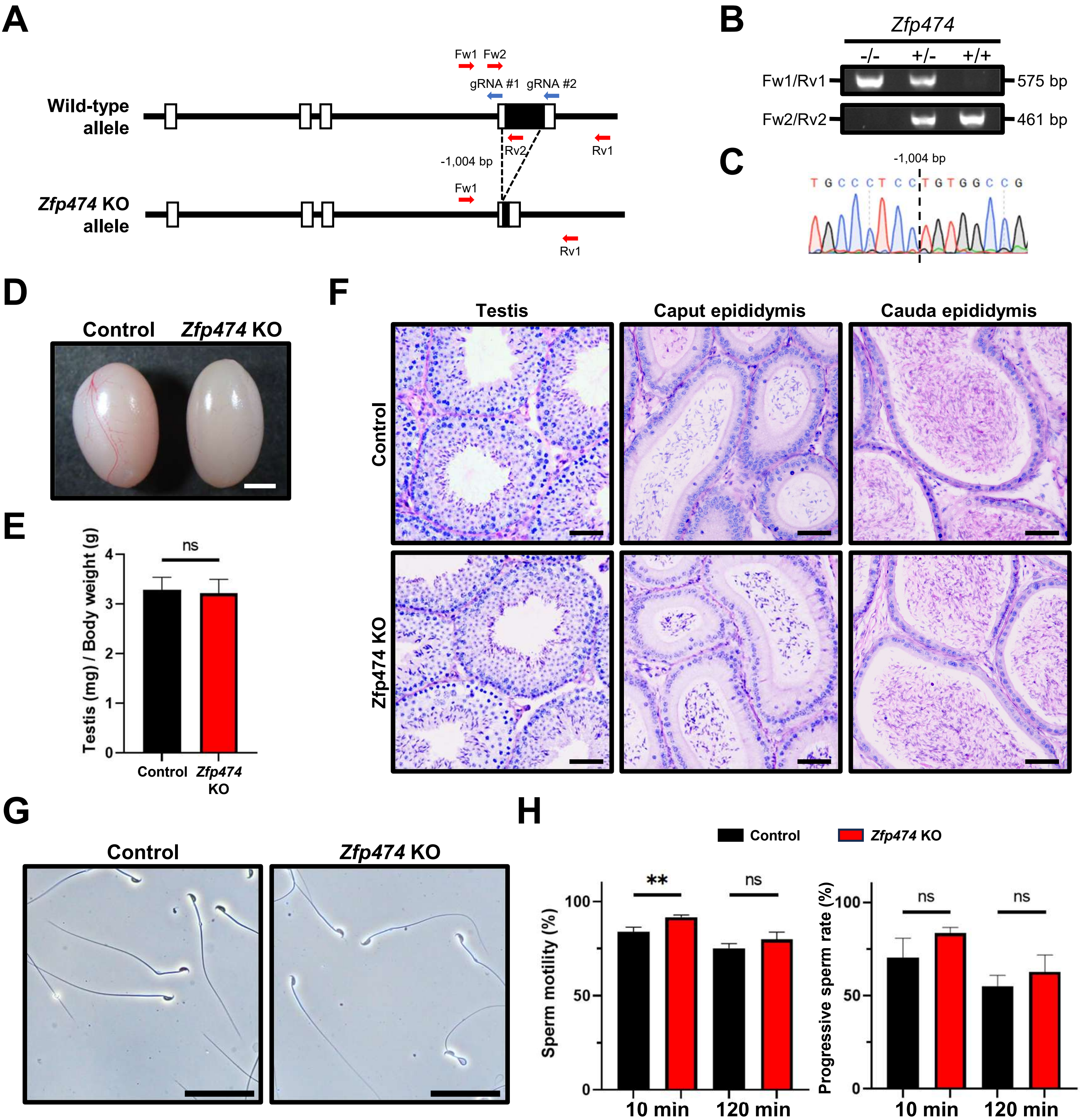

Figure S8. Phenotypic analysis of *Zfp474* KO male mice.
(A) KO strategy for generating *Zfp474* KO mice. The upper and bottom panels show diagrams for WT and KO alleles, respectively. Two gRNAs (blue arrows) were designed to remove almost the whole open reading frame. Fw1 and Fw2 are forward primers for genotyping. Rv1 and Rv2 are reverse primers for genotyping. Our study generated a *Zfp474* KO mouse line with a 1,004-bp deletion. (B) Genotyping of *Zfp474* KO mutant mice. Fw1/Rv1 and Fw2/Rv2 primers shown in Fig. S8A were used. (C) Sanger sequence of *Zfp474* KO mutant mice. The dashed line marks the deletion breakpoint. (D) Gross morphology of control and *Zfp474* KO testes. Scale bar: 2.0 mm. (E) The average testis weight per body weight of control and *Zfp474* KO mice (ns indicates not significant, Student's t-test, $N = 3$). (F) Hematoxylin and PAS-stained sections of the testes, caput, and cauda epididymides. Scale bars: 50 µm. (G) Morphology of spermatozoa from control and *Zfp474* KO mice obtained from the cauda epididymis. Scale bars: 50 µm. (H) Motile sperm rate and progressive sperm rate from control and *Zfp474* KO mice. Motility was checked after 10 min and 2 h of incubation in TYH media (** $P < 0.01$, ns indicates not significant, Student's t-test, $N = 3$).

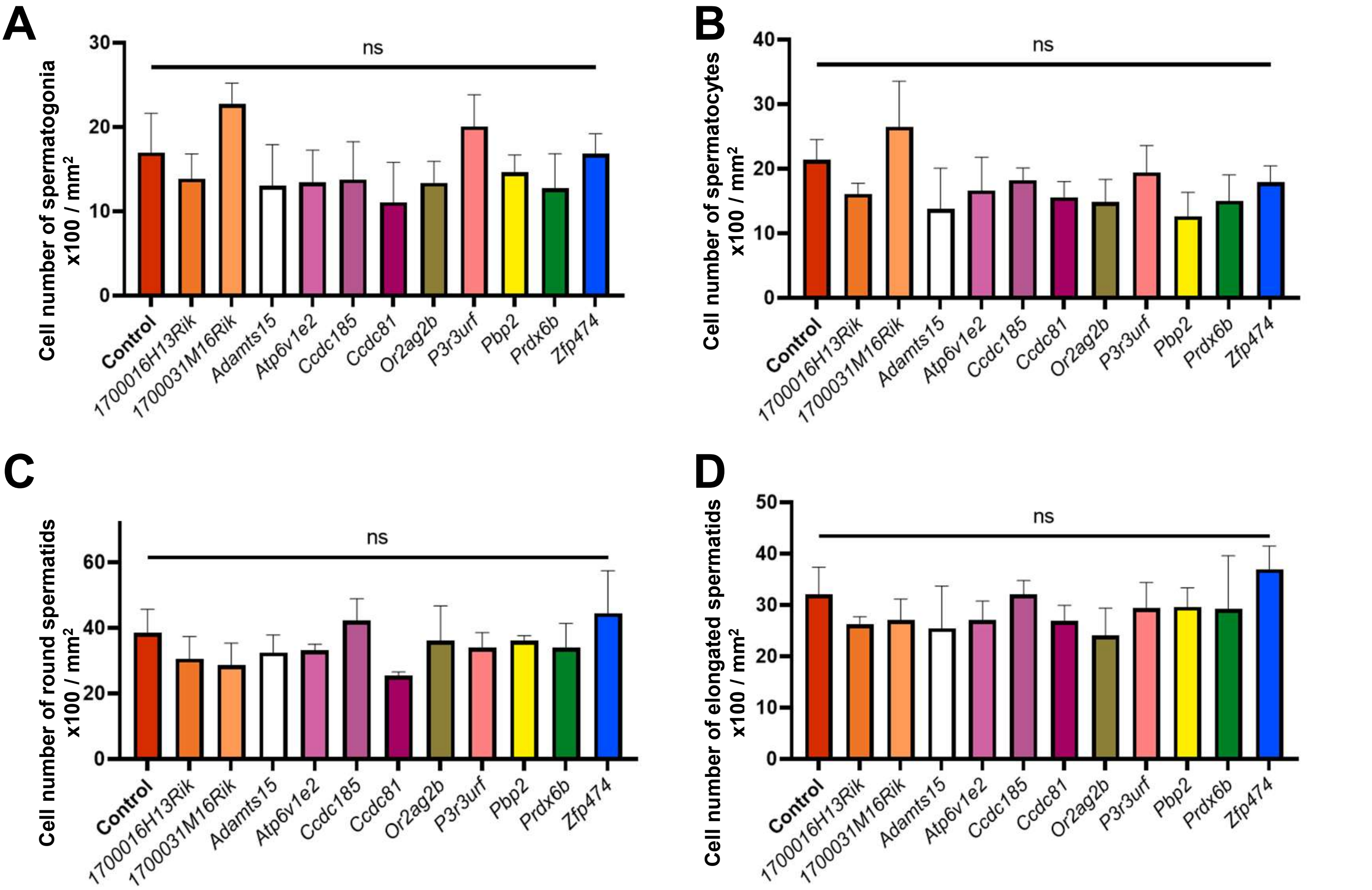

Figure S9. Germ cell number of stage VII seminiferous tubules.

(A) Cell number of spermatogonia from control and KO mice (ns indicates not significant, One-way ANOVA, *N* = 3).
(B) Cell number of spermatocytes from control and KO mice (ns indicates not significant, One-way ANOVA, *N* = 3).
(C) Cell number of round spermatids from control and KO mice (ns indicates not significant, One-way ANOVA, *N* = 3).
(D) Cell number of elongated spermatids from control and KO mice (ns indicates not significant, One-way ANOVA, *N* = 3).

Table S1. Primer list

Gene symbol	Primer set for WT	Annealing temperature (° C)	Cycles	Band size (bp)	Primer set for KO	Annealing temperature (° C)	Cycles	Band size (bp)
1700016H13Rik	Fw: GGCAGGCACTATTATGGGCT<br>Rv: TCCGATGGCTCAGGGGATAA	60	40	557	Fw: CCTCACCTTAGACTCTTACT<br>Rv: CCTTCATTTTGAGAAATCTC	60	40	544
1700031M16Rik	Fw: TTGGGTCTCGCTCTGAAGAT<br>Rv: CAAAGCAGACGTCCAGATGA	65	40	411	Fw: TTTGTGGACCATGTCTGTCC<br>Rv: TCCCAGGCTGCTTACTTTTG	65	40	637
Adamts15	Fw: GGCTCAGGTCCACCTCTTTC<br>Rv: GCCATACCCAAGGTGTCACA	60	40	597	Fw: CGACCGTACTAAAGCTCCCA<br>Rv: CTCCATTATGGGGGCCAAGG	60	40	601
Atp6v1e2	Fw: GCAGAGGCTGTAAGGAGGTG<br>Rv: ACATCTCACCTCCGGCAAAG	60	40	1,493	(same as WT)	60	40	706
Ccdc185	Fw: TCTCTGGTCTCCTTCCTCGG<br>Rv: ATCTCCGGGTTGTCAGCTTG	60	40	543	Fw: ATACACCACCCTTGTTTCCTAGAC<br>Rv: ACTGCACGTTCTTCTCTGGC	60	40	658
Ccdc81	Fw: AAGCCCTCTCTCCTCTCCAC<br>Rv: GCCACCAAGCTGGATCTAAG	60	40	553	Fw: CACCCTAAATTCTGGGCAAA<br>Rv: GATGGTCACTGAGGGAGGAA	60	40	528
Or2ag2b	Fw: AGTCACAGGCAGACTTCAGTC<br>Rv: AACATTAGCACCAATTTCCAACA	60	40	1,507	(same as WT)	60	40	412
P3r3urf	Fw: CCAAGGGATCTTGCTGTGGT<br>Rv: GGGCTGACATTGTCCACTGA	60	40	556	(same as WT)	60	40	465
Pbp2	Fw: TCCCAAGTCTTACCCACAAACA<br>Rv: GCCTGGTCTTTTCCCTTGGA	60	40	1,156	(same as WT)	60	40	701
Prdx6b	Fw: CCTGGAGCAAGGACATCAAT<br>Rv: CGGAGGTAATTTTTGCCAGA	60	40	415	Fw: GTGAATGTACACTCGGCAGG<br>Rv: GCACTCTGGGAGGCGAAAGG	60	40	599
Zfp474	Fw: ATTTCCAGCCAATCACAAGG<br>Rv: TGAGCCTGAGCACTCTGAAA	60	40	461	Fw: CCTCTAATGCATGGGTGGTT<br>Rv: AAAGGAGAGCCACTTGGACA	60	40	575

Table S2. Transembryo efficiency

Gene symbol	Guide RNAs	Embryo transfer efficiency	CRISPR/Cas9 efficiency	Sequence
1700016H13Rik	up: AGCGGTGCAGATGTTACATC<br>down: GAACCTGTTTCCGGCATCCA	3/30 (10.0%)	2/3 (66.7%)	-6,243 bp
1700031M16Rik	up: CTCTTCATTTCACCGACATG<br>down: TCATTGTCAAGTGACTACAG	10/47 (21.3%)	1/10 (10.0%)	-18,283 bp
Adamts15	up: AGGTGGTCGTTCCAATCCGA<br>down: GGCACCAGCACAACAGCGGG	2/55 (3.6%)	1/2 (50.0%)	-20,198 bp
Atp6v1e2	up: TTAACGTATCTACTCACGGA<br>down: GGAAAGTCGGCTGAATCTCG	8/22 (36.3%)	5/8 (62.5%)	-787 bp
Ccdc185	up: TGGCGCCGAGATCGAGACCG<br>down: AAACCGCTGCTTTGACCAGG	3/41 (7.3%)	1/3 (33.3%)	-1,708 bp
Ccdc81	up: TTCGGTCCCGATTGACTCCG<br>down: GTGCTATAGGGGCTGCGAAT	2/38 (5.3%)	1/2 (50.0%)	-37,018 bp
Or2ag2b	up: TCTTGTTGGCCATCCCACAA<br>down: GAGCTGTTAGAAGAGTACTG	13/46 (28.3%)	9/13 (69.2%)	-1,095 bp
P3r3urf	up: AAGTCAAGCCCGGAGGCCGA<br>down: AGTGTAGCCGCAGGACATAC	15/55 (27.3%)	4/15 (26.7%)	-91 bp
Pbp2	up: GCCGCTGAGCCTCCACGAAG<br>down: TGGTAACACGTGCCGGCCAC	6/55 (10.9%)	3/6 (50.0%)	-455 bp
Prdx6b	up: GTGGAAGCGGATACGGCCGA<br>down: TATGACGCTGTGATAGGCGG	10/44 (22.7%)	2/10 (20.0%)	-846 bp
Zfp474	up: TGACTCAGAGCGTCATTGGA<br>down: GCAAAAGTCCGGCCACAATT	9/49 (18.4%)	2/9 (22.2%)	-1,004 bp

Table S3. The ID of the mouse line used in this study

Gene symbol	Strain name	BRC number	CARD ID
1700016H13Rik	B6D2-*1700016H13Rik*^{em1Osb}	12432	3531
1700031M16Rik	B6D2-*1700031M16Rik*^{em1Osb}	12469	3568
Adamts15	B6D2-*Adamts15*^{em1Osb}	12467	3566
Atp6v1e2	B6D2-*Atp6v1e2*^{em1Osb}	12434	3533
Ccdc185	B6D2-*Ccdc185*^{em1Osb}	12453	3552
Ccdc81	B6D2-*Ccdc81*^{em1Osb}	12472	3571
Or2ag2b	B6D2-*Or2ag2b*^{em1Osb}	12261	3492
P3r3urf	B6D2-*P3r3urf*^{em1Osb}	12423	3523
Pbp2	B6D2-*Pbp2*^{em1Osb}	12433	3532
Prdx6b	B6D2-*Prdx6b*^{em1Osb}	12459	3558
Zfp474	B6D2-*Zfp474*^{em1Osb}	12464	3563

Table S4. Sperm motility analysis

Gene symbol	Genotype	Velocity Average Path (VAP; μm/sec)		Velocity Curved Line (VCL; μm/sec)		Velocity Straight Line (VSL; μm/sec)	
		10 min	120 min	10 min	120 min	10 min	120 min
Control	-	185.7 ± 3.7	170.5 ± 3.3	285.0 ± 5.7	270.4 ± 6.3	172.9 ± 3.3	151.2 ± 0.9
1700016H13Rik	-6,243/-6,243	166.6 ± 5.0	156.5 ± 9.1	244.8 ± 10.5	243.1 ± 15.6	154.4 ± 6.7	140.4 ± 8.2
1700031M16Rik	-18,283/-18,283	167.0 ± 8.1	158.0 ± 11.1	235.0 ± 13.5	234.6 ± 21.9	156.4 ± 6.8	145.1 ± 8.4
Adamts15	-20,198/-20,198	183.6 ± 19.9	150.4 ± 12.5	260.2 ± 20.6	240.6 ± 4.5	172.5 ± 20.9	134.2 ± 15.0
Atp6v1e2	-787/-787	148.0 ± 9.8	142.7 ± 13.5	262.0 ± 13.9	250.9 ± 23.0	126.0 ± 12.3	118.3 ± 0.7
Ccdc185	-1,708/-1,708	189.2 ± 3.8	155.7 ± 15.0	286.1 ± 14.4	244.4 ± 22.6	176.6 ± 4.9	139.4 ± 15.8
Ccdc81	-37,018/-37,018	151.2 ± 22.0	152.9 ± 18.2	253.9 ± 4.8	226.1 ± 1.5	132.3 ± 24.5	135.3 ± 20.6
Or2ag2b	-1,095/-1,095	167.5 ± 12.4	163.9 ± 6.9	250.2 ± 7.2	271.8 ± 7.5	154.1 ± 12.9	137.8 ± 6.6
P3r3urf	-91/-91	189.5 ± 16.8	160.8 ± 8.6	263.3 ± 14.3	243.2 ± 19.4	176.1 ± 18.2	146.1 ± 4.5
Pbp2	-455/-455	184.2 ± 9.0	182.5 ± 9.1	279.7 ± 14.0	275.1 ± 10.9	169.7 ± 14.0	166.4 ± 13.5
Prdx6b	-846/-846	157.5 ± 15.5	151.1 ± 6.6	255.1 ± 12.5	237.6 ± 7.6	143.1 ± 16.1	135.1 ± 7.9
Zfp474	-1,004/-1,004	182.3 ± 6.4	142.5 ± 12.2	275.0 ± 15.3	237.4 ± 6.3	167.3 ± 5.6	121.4 ± 12.7
